# The use of African medicinal plants in cancer management

**DOI:** 10.3389/fphar.2023.1122388

**Published:** 2023-02-14

**Authors:** Goabaone Gaobotse, Srividhya Venkataraman, Phenyo D. Brown, Kabo Masisi, Tebogo E. Kwape, David O. Nkwe, Gaolathe Rantong, Abdullah Makhzoum

**Affiliations:** ^1^ Department of Biological Sciences and Biotechnology, Faculty of Sciences, Botswana International University of Science and Technology, Palapye, Botswana; ^2^ Virology Laboratory, Department of Cell and Systems Biology, University of Toronto, Toronto, ON, Canada

**Keywords:** sub-Saharan Africa, medicinal plants, cancer, bioactive compounds, cancer management, anticancer mechanism

## Abstract

Cancer is the third leading cause of premature death in sub-Saharan Africa. Cervical cancer has the highest number of incidences in sub-Saharan Africa due to high HIV prevalence (70% of global cases) in African countries which is linked to increasing the risk of developing cervical cancer, and the continuous high risk of being infected with Human papillomavirus In 2020, the risk of dying from cancer amongst women was higher in Eastern Africa (11%) than it was in Northern America (7.4%). Plants continue to provide unlimited pharmacological bioactive compounds that are used to manage various illnesses, including cancer. By reviewing the literature, we provide an inventory of African plants with reported anticancer activity and evidence supporting their use in cancer management. In this review, we report 23 plants that have been used for cancer management in Africa, where the anticancer extracts are usually prepared from barks, fruits, leaves, roots, and stems of these plants. Extensive information is reported about the bioactive compounds present in these plants as well as their potential activities against various forms of cancer. However, information on the anticancer properties of other African medicinal plants is insufficient. Therefore, there is a need to isolate and evaluate the anticancer potential of bioactive compounds from other African medicinal plants. Further studies on these plants will allow the elucidation of their anticancer mechanisms of action and allow the identification of phytochemicals that are responsible for their anticancer properties. Overall, this review provides consolidated and extensive information not only on diverse medicinal plants of Africa but on the different types of cancer that these plants are used to manage and the diverse mechanisms and pathways that are involved during cancer alleviation.

## 1 Introduction

Over the past years, plants have gained momentum in research for the alleviation of major human diseases such as cancer and HIV/AIDS (Nkwe et al., 2021; Makhzoum and Hefferon, 2022). Plants have been used in the production of anti-HIV recombinant proteins to circumvent issues associated with the use of antiretroviral drugs (Tremouillaux-Guiller et al., 2020; Gaobotse et al., 2022a). Cancer, like HIV/AIDS, is a major cause of global morbidity and mortality. The World Health Organization (WHO) reported that cancer is the second leading cause of death globally and was responsible for an estimated 9.6 million deaths in 2018 (WHO, 2020). Approximately 70% of deaths from cancer occur in low and middle-income countries which are mostly in Africa. It is estimated that 13% of global annual deaths are due to lung, colorectal, stomach, liver, and breast cancers (WHO, 2020). It is also estimated that by the year 2030, cancer related deaths will increase to 17 million (Adeloye et al., 2016). According to (WHO, 2020), in Africa, the prevalence of cancer is higher in men than women. More than 19.3 million new cases of cancer were registered worldwide in the year 2020 and these numbers are expected to rise to 28.4 million by the year 2040 (Sung et al., 2021). In the same year of 2020, there were 10 million reported deaths due to different types of cancers across the world. In addition, female breast cancer has now surpassed lung cancer as the most diagnosed cancer in the world (Sung et al., 2021). However, lung cancer remains the leading cause of mortality amongst all the different types of cancer, causing an estimated 1.8 million deaths in the year 2020. This is equivalent to 18% of all cancer related deaths globally. On the other hand, female breast cancer contributed to only 6.9% of all global cancer related deaths in 2022 (Sung et al., 2021). Throughout history, human beings have acquired knowledge on the medicinal uses of plants and have applied this knowledge in folk medicine to treat different diseases (El-Seedi et al., 2013; Ouelbani et al., 2016). It is estimated that 80% of the world population still rely on plant-based material for primary healthcare while traditional medicine usage accounts for 60% of the world population (El-Seedi et al., 2013). Due to low income or long distances from urban treatment centers, many people in Africa commonly use medicinal plants for cancer treatment (Kabbaj et al., 2012; El-Seedi et al., 2013). Moreover, some Africans believe that medicinal plants are more effective than synthetic drugs in managing diseases such as cancer. However, comprehensive compilation of information on these medicinal plants of Africa is insufficient. The documented use of medicinal plants in Africa dates back a couple of centuries, although it might have been there earlier than that (Sawadogo et al., 2012; Ntie-Kang et al., 2013). Cancer remains one of the leading causes of death globally despite advancements in cancer management strategies. Although there have been recent efforts in the production of cancer vaccines in different host plants for different types of cancer (Gaobotse et al., 2022b), there is a need to explore medicinal plants further for cancer management strategies. This review is a compilation of information on diverse medicinal plants of Africa, found in different countries of the continent, that have been used in the management of different types of cancer.

Cancer is the third leading cause of premature death in sub-Saharan Africa, responsible for one in 7 deaths. In 2020, there were 801, 392 new cases of cancer with an estimated 520, 158 deaths in sub-Saharan Africa. In women, the most common types of cancer are breast (129, 400) and cervical cancer (110, 300) which together are responsible for three out of 10 cancer diagnoses. In men, the most common types of cancer are prostate (77, 300), liver (24, 700) and colorectal cancer (23, 000) (Bray et al., 2021; Bray et al., 2022).

The rich flora of Africa results from a vast difference in environmental and climatic conditions such as deserts, savannah, and tropical rain forests. Some of these plants have been used in traditional medicine to treat symptoms of cancer with reports that of all pharmaceutical drugs used, a quarter of them have been derived from plants originally used in traditional medicine (Sawadogo et al., 2012). Treatment of patients across Africa varies from one region to another and this is mostly influenced by the plants, protocols and recipes used. In West Africa, more than half of plants with anticancer metabolites are shrubs and the explants commonly used are the roots and stem barks, with methanol extracts contributing approximately 60% of the used extracts (Sawadogo et al., 2012). (Sawadogo et al., 2012) have shown that phytochemicals with high cytotoxicity towards many cancer cell lines are diterpenes, triterpenes and steroids. Leukaemia, breast, colon, and lung cancer cell lines have shown great sensitivity toward phytochemicals isolated from plants with anticancer activity in West Africa (Usman et al., 2022). *Brassica rapa*, which is found across almost all regions of Africa, has phytochemicals such as phenanthrene, diarylheptanoids and others with cytotoxic activity towards various human cancer cell lines (Wu et al., 2013). The African cabbage has been used by traditional healers to treat tumors and its extracts have been shown to be cytotoxic to carcinoma cell lines in mice (Bala et al., 2010). *Colocasia esculenta,* a tropical plant found in most regions of Africa, is said to produce fibers which antagonize the growth of colon cancer cells in rats (Brown et al., 2005) and its aqueous extracts inhibit breast and lung tumors (Kundu et al., 2012).

Twenty-three (23) anticancer plants that are native to Africa will be discussed in this review. These plants are *Dicoma anomala*, a perennial herb; *Fagaropsis* which are shrubs or deciduous trees with buttress roots; *Tribulus terrestris*, which is a small, silky, and hairy herb; *Portulaca oleracea*, which is an annual succulent plant and *Withania somnifera*, a small, bushy, evergreen shrub. Other plants that will be discussed are the semi-deciduous tree called *Azanza garckeana*; *Cajanus cajan*, which a perennial legume; *Combretum caffrum*, which is an African bush willow tree; and the flowering cherry plants called *Prunus avium* and *Prunus africana*. The review will also look at the tree species *Securidaca longipedunculata*; the shrub *Annona senegalensis*; the tropical fruit tree *Annona muricata*; the shrub plant *Aerva javanica* and the flowering plant *Abelmoschus esculentus*. The review will also discuss the dioecies *Flueggea virosa*; the climbing vine called *Lagenaria siceraria*; the aromatic evergreen called *Xylopia aethiopica*; the flowering perennial aquatic *Nymphaea lotus* as well as the deciduous shrub called *Zanthoxylum chalybeum*. The Mediterranean evergreen *Ceratonia siliqua*; the perennial softwood vegetable *Moringa oleifera* and the perennial herbaceous *Peganum harmala* will also be discussed in the review.

Amongst these 23 plants, *T. terrestris* can be consumed in diet. The leaves, fruits, and shoots of this plant can be cooked and consumed. *Portulaca oleracea* is an edible weed with high nutrition. It also has nutraceutical value that helps in the prevention, treatment, and management of some human diseases (Gunarathne et al., 2022). In India, *Withania coagulans* is used to ferment milk during cheese production (Ahmad et al., 2017). *Azanza garckeana* is a valuable source of fruits which are consumed while green or a bit ripe (Ochokwu et al., 2015). *Cajanus cajan* is a good source of protein and its seeds are cooked and consumed as peas (Talari and Shakappa, 2018). *Prunus avium* fruits comprise of a fleshy edible mesocarp as well as an edible protective exocarp (Usenik et al., 2015). However, the endocarp of the fruit is inedible. The leaves of *S. longipedunculata* are cooked and consumed while they are young while the roots of this plant are poisonous. The ripe fruits of *A. senegalensis* are edible while the flowers of the plant are used for food seasoning (Donhouedé et al., 2022). The fruits of *A. muricata* are also consumed raw or cooked. *Abelmoschus esculentus* is an important vegetable crop whose immature fruits can be consumed as vegetables (Gemede et al., 2015). The fruits of *F. virosa* are only edible when mature. The fruits, seeds, leaves, and oil of *L. siceraria* are edible (Zahoor et al., 2021). The tubers of *N. lotus* are consumed raw or roasted (Lim, 2014). The leaves of *Z. chalybeum* are dried and used as powdered vegetable (Balama et al., 2015).

## 2 Traditional African medicinal plants used in the management of different cancers

### 2.1 *Dicoma anomala*

*Dicoma anomala* is a perennial herb that is typically called stomach or fever bush. It is a member of the family Asteraceae. It has an erect stem covered with thin hairs as well as an underground tuber (Balogun and Ashafa, 2017). It is native to sub-Saharan Africa, and, in South Africa, it is widely distributed in the Free State, Gauteng, KwaZulu-Natal, Limpopo, Mpumalanga, Northern Cape, and North West provinces. This plant has been used traditionally in Africa to treat diseases such as colds, coughs, fever, ulcers, and diabetes (Balogun and Ashafa, 2017). It has wide ethnomedical uses and its roots are universally used to treat diseases that affect animals and humans. Among the different species of *Dicoma, D. anomala, D. zeyheri, D. capensis and D. schinzii* have been utilized for their medicinal properties and were duly classified based on their phytochemical composition. Some of these phytochemicals are acetylenic compounds, flavonoids, phenolic acids, phytosterols, sesquiterpenes and triterpenes. They are non-toxic to normal non-cancerous cells. These compounds occur primarily in the leaves and roots and have been used to treat various cancers such as prostate, kidney, ovarian and breast cancers (Greenwell and Rahman, 2015; Maroyi, 2018). *D. anomala Sond* roots have shown antiproliferative effects against MCF-7 breast cancer cells wherein sesquiterpene conjugated to silver nanoparticles demonstrated anticancer properties by causing oxidative damage in the cancerous cells (Shafiq et al., 2020). *Dicoma capensis* aqueous extracts have also shown anticancer properties against breast cancer cell lines such as MCF-12A, MDA-MB-231 and MCF-7 (Asita et al., 2015).

### 2.2 *Fagaropsis*

The genus *Fagaropsis* belongs to the family Rutaceae and is widely distributed in Africa. *Fagaropsis* are mostly shrubs or deciduous trees with buttress roots. The plant species *Fagaropsis angolensis* is found in Angola, Ethiopia, Kenya, Namibia, Tanzania, Uganda, and Zimbabwe; *F. hildebrandtii* is primarily found in Ethiopia, Kenya, Somalia, and Tanzania while *F. velutina* and *F. glabra* occur endemically in Madagascar (Sun et al., 2021). *F. angolensis* whole root methanol extracts have been shown to exhibit antitumor activity against throat cancer cells (Hep2) at an IC_50_ value of 10.05 ± 2.15 μg/mL. Whole root and root-stem methanol extracts showed high degrees of anticancer effects against CT26 colon cancer cells with IC_50_ values of 8.33 ± 1.42 μg/mL and 5.25 ± 0.35 ug/mL respectively (Yiaile, 2017). *F. angolensis* stem bark methanolic extracts demonstrated significant activity against DU-145 prostate cancer cells and HCC 1395 breast cancer cells at IC_50_ values of 12.8 ± 1.1 μg/mL and 53.9 ± 5.6 μg/mL respectively (Misonge et al., 2019). Anticancer effects of methanol extracts of the bark of *F. angolensis* against HCC 1395 cells were also reported by (Onyancha et al., 2018). Despite its anticancer activities, *F. angolensis* has shown high levels of toxicity on Vero cells. Therefore, more research is needed to evaluate the precise dosages of the active anticancer ingredients in this plant to mitigate toxicity issues and validate them for their use in folklore medicine.

### 2.3 *Tribulus terrestris*

*Tribulus terrestris* is a small, silky and hairy herb which is indigenous to tropical regions, including Africa, and is a member of the Zygophyllaceae family (Hashim et al., 2014). Methanol extracts of *T. terrestris* showed strong inhibition against SK-OV-3 ovarian carcinoma cells and MCF-7 breast cancer cells with IC_50_ values of 89.4 μg/mL and 74.1 μg/mL respectively (Abbas et al., 2022). *Tribulus terrestris* occurs as a perennial herb and is known for its antineoplastic effects against a wide range of human cancers. The high anticancer potential of *T. terrestris* has been attributed to its high content of steroidal saponins which have been shown to induce programmed cell death in MCF-7s by eliciting both extrinsic and intrinsic apoptotic pathways (Osbourn, 2003; Sun et al., 2003; Sparg et al., 2004; Kim et al., 2011; Angelova et al., 2013; Faizal and Geelen, 2013). (Patel et al., 2021) reported *in silico* studies implicating the anticancer properties to active saponin compounds such as nuatigenin saponin that could be essential in the treatment of breast cancer. *T. terrestris* fruit extracts are capable of inhibiting autophagy in TW2.6 and SAS oral cancer cells and can impact cell proliferation, growth, cell migration and invasion of neoplastic/metastatic cancer cells (Shu et al., 2021).

### 2.4 *Portulaca oleracea*

*Portulaca oleracea* is a common succulent plant that is often referred to as Purslane. It belongs to the genus *Portulaca* in the family Portulacaceae and is abundant in most regions of Africa such as Botswana (Uddin et al., 2014). The polysaccharides of *P. oleracea* L., POL-P3b, have shown inhibition of tumors by means of cell cycle arrest, elicitation of DNA damage and induction of apoptosis (Zhao et al., 2013). (Jia et al., 2021) demonstrated that there was a high-level induction of TNF-α, IFN-γ and IL-12 when POL-P3b was co-administered with a dendritic cell vaccine in mouse models, implicating Th1 immune response modulation by POL-P3b. Therefore, POL-P3b serves as an adjuvant that stimulates maturation and augment the antigen presentation capability of the Dendritic Cell (DC) vaccine. Additionally, POL-P3b upregulated the expression levels of MyD88, NF-κB and TLR4 in the DC vaccine and promoted Th1 cytokine secretion. The Ki 67 index is closely associated with the degree of tumor malignancy (Bleckmann et al., 2016; Christensen et al., 2016) and a combination of the DC vaccine and POL-P3b was found to be capable of reducing Ki 67 expression. Also, this combination triggered more significant apoptotic characteristics such as nuclear fragmentation, massive shrinkage of cells and high numbers of TUNEL-positive cells when compared to either the DC vaccine alone or POL-P3b alone. Additionally, higher immunomodulatory properties were observed in immune mice when administered with POL-P3b (Jia et al., 2021). Lymphocyte proliferation elicited by LPS and Con A was augmented, the CD4+/CD8+ ratio increased and the expression of cytokines, including that of TNF-α, IFN-γ, IL-4 and IL-12p70, was also significantly enhanced, suggesting that the POL-P3b adjuvant administered along with the vaccine can upregulate immune response reactions in mice. CD31, CD34 and VEGF expressions were more pronouncedly diminished when a combination of POL-P3b and the DC vaccine was administered and there was a synergistic suppression of angiogenesis. POL-P3b as an adjuvant for the DC vaccine also demonstrated diminished mortality and prolonged survival of tumor-bearing mice, showing the anti-tumor effect of POL-P3b. Therefore, POL-P3b functions as a highly propitious dietary adjuvant for the DC vaccine, inducing DC maturation. POL-P3b, in addition to inhibiting tumor growth by increasing tumor apoptosis, also inhibited lung metastasis, and induced non-toxic side effects in mouse models (Jia et al., 2021). Therefore, POL-P3b holds great promise as an efficient and safe immunomodulatory agent capable of regulating DC maturation and enhancing immune responses of the DC vaccine against breast cancer (Liu et al., 2021). isolated two novel amide glycosides, oleraciamide E and oleraciamide F, containing similar molecular structures from *P. oleracea* L. wherein oleraciamide E demonstrated anticholinesterase activity with an IC_50_ value of 52.43 ± 0.33 M and exhibited scavenging activity in 1,1-diphenyl-2-picrylhydrazyl (DPPH) radical quenching assays with an IC_50_ value of 24.64 ± 0.33 M which indicate antioxidant effects.

### 2.5 *Withania somnifera*

*Withania somnifera* is a small, bushy, evergreen shrub widely distributed across the world and abundant in South Africa and Botswana. It is a member of the Solanaceae family (Gaurav et al., 2015). It has been shown that *W. somnifera* root extracts can inhibit vimentin, a protein normally found in regions of metastasis, thus suggesting its counteracting effects on tumour formation in breast cancer (Yang et al., 2013). The anticancer potential of *W. somnifera* has been mainly attributed to Withanolide and Withaferin A, which are two principal phytochemicals from this plant. *In vivo*, in mice, it was reported that *W. somnifera*, through the activities of Withanolide and Withaferin-A, modulate different signalling pathways such as apoptosis, autophagy and reactive oxygen species (ROS) pathways (Shikder et al., 2020). In male Swiss albino mice, extracts from *W. somnifera* were shown to inhibit lung adenoma (Senthilnathan et al., 2006a) while the root extracts of this plant were shown to prevent ROS-induced injury in model mice (Senthilnathan et al., 2006b). Ethanol extracts of the roots of *W. somnifera* were shown to inhibit the proliferation of A549 lung cancer cells through the downregulation of PI3K, which reduced metastasis (Vamsi et al., 2020). *In vitro*, Withaferin-A has been shown to reduce the proliferation of the human breast cancer cell line MDA-MB-231 by inhibiting the two-pore domain potassium (K2P9) channel TASK-3 (Zúñiga et al., 2020). Withaferin-A has also been shown to inhibit the proliferation of other cancer cell lines such as the cervical cancer cell lines (HeLa, SKGII, ME180) and ovarian cancer cell lines (OKV-18 and SKOV3) (Sari et al., 2020). The activity of Withaferin-A against these cells is said to be brought about by the upregulation of the tumour suppressor p53 coupled with cell growth arrest and DNA damage signalling (Sari et al., 2020). Withanolides have been shown to inhibit the proliferation of MCF-7s by inducing apoptosis, inducing the overexpression of Hsp70 and reducing the expression of ER in MCF-7s (Chen et al., 2008). Withanolides were also found to suppress TGF-b1 and TNF-a induced Epithelial-Mesenchymal Transition (EMT) in the lung cancer cell lines H129 and A549 (Yang et al., 2013). Table 1 shows bioactive compounds of *Withania*, the plant parts they are derived from, the different types of cancer that they target, and their mechanisms of action during cancer alleviation.

**TABLE 1:** *Withania* bioactive compounds, plant parts they are derived from, their target cancers, and their mechanisms of action.

Bioactive molecules	Parts used	Cancer cell line/Experiment	Mechanism of action
Withaferin A (WA)	Leaves	Breast cancer cell lines; MCF-7 and MDA-MB-231	Inhibited the expression of ER, HSF1 and RET; increased the expression of p21, phospho-p38 MAPK, and p53 in MCF-7s Zhang et al. (2011)
		Mouse models and breast cancer cells	Blocked cell proliferation, diminished tumor growth and promoted FOXO3a and Bim-dependent apoptosis Stan et al. (2008)
		Ovarian cancer cells	Blocked cell growth, induced cell cycle arrest and apoptosis, targeted Notch1 and Notch3 downregulation Zhang et al. (2012)
		Breast cancer cell lines; 4T1 (mouse breast), SCID mice, Balb/c mice, Nu/nu mice	Decreased tumor growth and enabled chemoprevention Thaiparambil et al. (2011)
	Roots	Prostate PC-3 xenografts in nude mice	Inhibited proteasomal chymotrypsin-like activity and tumor growth Yang et al. (2007)
		Breast cancer cell lines; SUM159 and MCF-7	Induced apoptosis and showed antiproliferative activity Hahm et al. (2014)
	Fruits	Liver cancer cells HepG2	Notably changed chromatin structure (uniform condensation, fragmentation) Abutaha (2015)
L-asparaginase	Fruits	Human leukemia cells	Inhibited lymphoblastic leukemia Oza et al. (2010)
Withaferin A and Withanolide D	Roots	B16F-10 melanoma cells in C57BL/6 mice	Showed significant antitumor activity Leyon and Kuttan (2004)
Withania somnifera	Leaves	Human glioma cell lines (A172, YKG1 and U118MG)	Inhibited cell proliferation and increased the expression of NCAM and mortalin Kataria et al. (2011)
Withania somnifera and Withaferin	Leaves	Breast carcinoma (MCF-7), human normal fibroblasts (TIG-3) and colon carcinoma (HCT116)	Enhanced DNA damage and oxidative stress; downregulated ING1, LHX3, TPX2 and TFAP2A Widodo et al. (2010)
Withania somnifera	Roots	Prostate cancer cells (PC-3)	Inhibited cell proliferation and arrested cell cycle in G2/M phase; downregulated the expression of COX-2 and IL-8 Balakrishnan et al. (2017)
Withania somnifera and cisplatin	Roots	Colon (HT-29) cancer cells and breast (MDA-MB-231) cancer cells	Inhibited cell proliferation, promoted mitochondrial dysfunction, and generated ROS Henley et al. (2017)

### 2.6 *Azanza garckeana*

**FIGURE 1:**
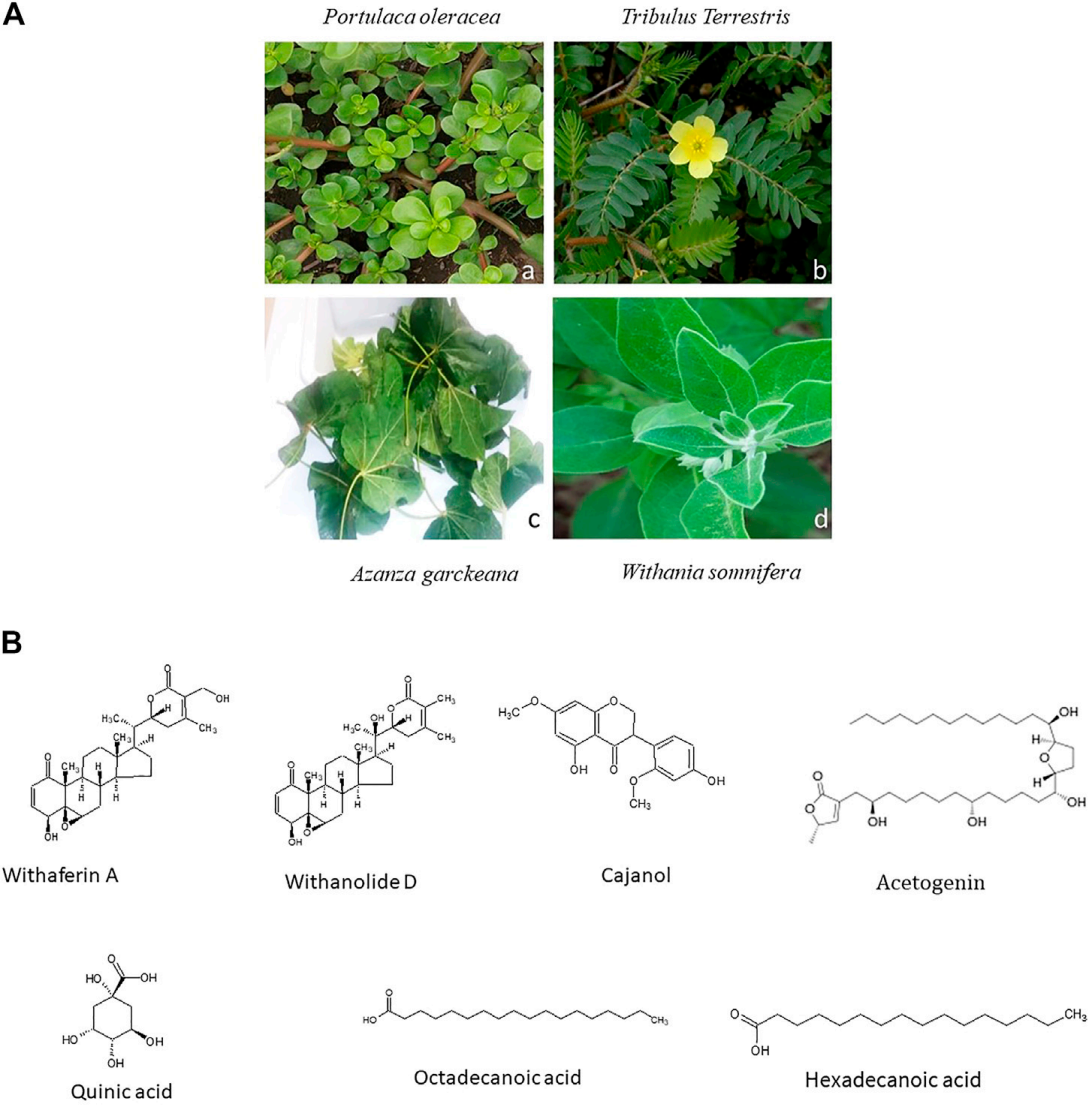
**(A)** Images of a selected African Medicinal Plants species used for Cancer management. Leaves of *Portulaca oleracea* are fleshy and oval **(a)** (Source: https://www.terrepromise.ca/en/products/lettuce/greens/[accessed 12 Oct 2022]), *Tribulus Terrestris* leaves are opposite each other and unequal in size **(b)** (Source: https://www.zimbabweflora.co.zw/speciesdata/image-display.php?species_id=132940&image_id=4 [accessed 3 Nov 2022])*,* Harvested leaves of *Azanza garckeana* showing a linear midrib fissure **(c)** *, Withania somnifera* leaves are broadly ovate with curvy margins **(d)** (Source: https://www.magicgardenseeds.co.uk/The-Good-To-Know/Winter-Cherry-(Withania-somnifera)-A.WIT01- [accessed 27 October 2022]). **(B)** Structures of some Phytochemicals derived from African Medicinal Plants.

*Azanza garckeana* is a semi-deciduous tree and a member of the Malvaceae family that is commonly found in East and Southern Africa. According to (Michael et al., 2015), on evaluating phytochemicals of the seeds of this plant, it was discovered that they have tannins, saponins, flavonoids, alkaloids, phenols, glycosides and carotenoids. The benzopyrone ring structure, known for its antioxidant properties, is integral to flavonoids, making flavonoids to have antioxidant behaviour. This antioxidant activity is able to fight and remove free radicals in biological systems and inhibit the development of tumors (Michael et al., 2015). The seeds of *A. garckeana* are said to be able to reduce cancer development due to their possession flavonoids, which are able to interfere with oestrogen synthase, an enzyme that produces oestrogen (Okwu, 2005). A complex formed between Mansone G and β-Cyclodextrin, which are some of the phytochemicals extracted from *A. garckeana*, was found to exhibit high levels of cytotoxicity towards A549 lung cancer cells (Bioltif et al., 2020). Figure 1 shows the leaves of *Portulaca oleracea, Tribulus terrestris*, *A. garckeana and Withania somnifera.*

### 2.7 *Cajanus cajan*

Ovarian cancer is the second most prevalent gynecological malignancy and one of the most lethal cancers. The major challenge in the treatment of ovarian cancer is the emergence of multi-drug resistance during chemotherapy. Cajanol is derived from *Cajanol cajan* roots and has a multitude of pharmacological activities such as anti-tumor properties. Cajanol inhibits NF-κB phosphorylation and nuclear ectopia by interfering with PI3K expression and the phosphorylation of Akt. This diminishes the transcription and translation of the permeability glycoprotein and eventually decreases cancer related multi-drug resistance that is induced by the efflux of paclitaxel (Sui et al., 2021). *Cajanus cajan* is a medicinal plant that is native to the Southwest of Nigeria. It is grown for food in Nigeria (Ashidi et al., 2010). *C. cajan* synthesizes cajanin stilbene acid (CSA) which shares structural analogies with estrogen. CSA exerts antiestrogenic and anticancer activities towards estrogen receptor (ERα)-positive breast cancer cells. Particularly, it shows cytotoxicity towards MCF-7 cells resistant to tamoxifen while exhibiting low cytotoxicity towards ERα-negative breast tumor cells (Fu et al., 2015). This cytotoxicity is independent of the cellular p53 status. CSA binds to the same site as tamoxifen and 17β-estradiol on ERα. Thus, CSA displays its anticancer properties against ERα-positive breast cancer cells by interacting with and inhibiting ERα. The cytotoxicity of CSA towards MCF-7s resistant to tamoxifen indicates its promise as a tamoxifen alternative for treatment of breast cancer. When combined with tamoxifen, CSA enables synergistic cytotoxicity and promotes p53 protein expression. Cajanol, scientifically known as 5-hydroxy-3-(4-hydroxy-2-methoxyphenyl)-7-methoxychroman-4-one, is an isoflavone isolated from the roots of *C. cajan*. In MCF-7s, cajanol causes cell cycle arrest at the G2/M phase while inducing apoptosis through a mitochondrial pathway mediated by ROS (Luo et al., 2010). This leads to the disintegration of the outer membrane of the mitochondria and the release of cytochrome C followed by the elicitation of the caspase-3 and caspase-9 cascade which causes apoptosis. In COR-L23 lung cancer cells, *C. cajan* was shown to have anticancer activity with an IC_50_ value of 5–10 μg/mL. It was reported that stilbenes longistylins A and C present in the leaf extracts of the plant were responsible for this anticancer property (Ashidi et al., 2010).

### 2.8 *Combretum caffrum*

The bark of *Combretum caffrum*, the African bush willow tree, is a source of natural phenolic stilbene compounds called combrestatins (Pettit et al., 1987). Among these combrestatins, combrestatin A-4 (CA4) is the most efficient as an antitumor agent. CA4 inhibits tubulin polymerization, augments vascular permeability, and abrogates blood flow into tumors (Simoni et al., 2006). A drug candidate, Ecust004, has been developed as an optimized agent derived from the structure-activity investigations of the sulfamate derivatives of CA4 and Erianin. This is a strong inhibitor of steroid sulfatase and tubulin in addition to exhibiting antiproliferative activity against tumor cells at low concentrations. This sulfate modification augments the pharmacokinetic profiles and bioavailability of the parental CA4 and Erianin natural compounds (Raobaikady et al., 2005; Foster et al., 2008; Visagie et al., 2015). Ecust004 inhibits tumor proliferation *in vivo* and *in vitro* in addition to diminishing cell viability, migration, and invasion of MCF-7 and MDA-MB -231 cells at low dosages. Combrestatins comprise a category of closely analogous stilbenes including combretastatins A, dihydrostilbenes or combretastatins B, phenanthrenes or combretastatins C and macrocyclic lactones or combretastatins D which are all derived from the bark of *C. caffrum*. Some of these compounds are the strongest antitubulin agents ever known. Owing to the structural simplicity of these substances, several analogs have been synthesized. Amongst these, Combrestatin A4 phosphate is the most tested substance in preclinical and clinical trials (Karatoprak et al., 2020). This water-soluble prodrug is rapidly metabolized in the body to combrestatin A4 that displays antitumor, antiproliferative, anti-inflammatory and antioxidant activities. Nano-formulations of CA4 phosphate have important advantages such as increased water solubility, drug targeting capabilities, improved efficiency, protracted half-lives in circulation and lesser side effects. Therefore, combrestatins are favorable candidates for novel tumor therapeutics.

Combrestatins have been shown to be active against many human cancer cell lines and combrestatin A-4 is the strongest in terms of potency. Their principal action is their interaction with the colchicine binding site of the tubulin b subunit and the disruption of tubulin polymerization into microtubules (Izuegbuna, 2022). CA4 has been shown to be cytotoxic towards some cancer cell lines such as the leukemia cell line, P-388, which is resistant to daunorubicin (McGown and Fox, 1990). It acts by disrupting cell signaling pathways involved in the maintenance and regulation of the cytoskeleton of the endothelial cells occurring in the tumor vasculature, thus causing the selective disruption of blood flow through the tumors. Thereupon, these tumor cells undergo necrosis. Intriguingly, while enabling disruption of blood supply into the tumor cells, CA4 maintains normal blood flow into adjacent normal tissues (Nihei et al., 1999). CA4 activity is not dependent on temperature. This contrasts with colchicine, which interacts with a similar binding site on the tubulin molecule. The complexation of CA4 tubulin occurs easily even on ice (Nam, 2003). Another CA4 sodium phosphate (CA4P) pro-drug has also been synthesized. The action of CA4P is like that of CA4. It leads to microtubule depolymerization and represses tumor cell angiogenesis (Tozer et al., 2002). CA4P eventually induces apoptosis in tumor cells (Iyer et al., 1998), although it has been reported that it results in cell death through molecular pathways, rather than apoptosis, such as the mitotic catastrophe pathways (Nabha et al., 2001). These pathways, which lead to cell death, make combrestatin a powerful anticancer compound of high efficacy (Jaroch et al., 2016).

### 2.9 *Prunus avium*

*Prunus avium* (Sweet cherry) is a plant that is widely distributed in the north of Africa (Osafo et al., 2017). Dark sweet cherry is a rich source of phenolics and is characterized by its anti-invasive and anticancer activities. *Prunus avium* phenolics inhibit MDA-MB-453 breast cancer cells by triggering cell signaling pathways that elicit apoptosis and disrupt cell invasion. Amongst these phenolics, anthocyanins have shown augmented chemopreventative properties (Layosa et al., 2021). Polyphenols, especially anthocyanins, are well known for their anti-inflammatory, cytoprotective and antioxidant properties (Tsuda et al., 2000; Li et al., 2014; Huang et al., 2018; Silva et al., 2020). Table 2 shows examples of anticancer bioactive compounds found in *P. avium* and outcomes of their experimental uses.

**TABLE 2:** Biological properties of *Prunus avium* extracts and their bioactive compounds that target hallmarks of cancer.

Cancer hallmark	Biological model/Type of study	Experiment	Outcome
Oxidative stress	*In vitro*: Hep2G cells	Incubation with extract of sweet cherries	↓ Intracellular ROS Acero et al. (2019)
	*In vitro*: Caco-2 cells	Co-incubation with sweet cherry extract and H_2_O_2_	↓ Carbonyl proteins
			↓ Intracellular ROS restored GSH/GSSG ratio Matias et al. (2016)
	*In vitro*: SH-SY5Y cells	Pre-incubation with sweet cherry extract prior to H_2_O_2_ administration	↓ Intracellular ROS
			↑ GSH ↑ GR ↑ NQO1 Antognoni et al. (2020)
	*In vivo*: Wistar rats	High fructose-diet with freeze-dried sweet cherry	↑ GPx ↑ GR
			↓ Catalase ↓ SOD
			Inhibition of lipid peroxidation Dziadek et al. (2019)
	*In vivo*: Human subjects	Consumption of sweet cherries	↑ Plasma lipophilic antioxidant capacity
			↑ Plasma hydrophilic antioxidant capacity
			↑ Urinary antioxidant capacity
			↑ Lipophilic oxygen radical absorbance capacity
			↓ Ferric reducing ability of plasma Kelley et al. (2006); Prior et al. (2007); Garrido et al. (2010)
Inflammation	*In vivo*: Wistar rats	High fructose-diet along with freeze-dried sweet cherry	↓ CRP ↑ IL-10 Dziadek et al. (2019)
	*In vivo*: Diet-induced obese mice	Diet supplemented with cyanidin-3-glucoside, cyanidin-3-rutinoside and pelargonidin-3-glucoside extracted from sweet cherries	↓ IL-6
			↓ Inducible NO synthase
			↓TNF-α ↓ NF-κB (Wu et al., 2016)
	*In vivo*: Human subjects	Daily consumption of sweet cherries	↓ CRP ↓ EGF ↓ Endothelin 1 ↓ EN-RAGE ↓ Ferritin ↓ IL-18 ↓ PAI-1 ↑ IL-1 receptor antagonist ↓ Ferritin Kelley et al. (2013)
	*In vitro*: HeLa cells	Incubation with sweet cherry crude extract	↓ Cell viability Pacifico et al. (2014)
Cell death and proliferation	*In vitro*: MKN45 cells	Incubation with sweet cherry extract	↓ Cell viability Serra et al. (2011)
	*In vivo*: MDA-MB453 cells xenograft mice model	Oral administration of sweet cherry whole extract	↓ Tumor growth
			↑ Phosphorylated ERK 1/2
			↓ AKT ↓ STAT3 ↓ p38-MAPK ↓ JNK ↓ NF-κB ↓ Ki-67 Noratto et al. (2020)
Invasion and metastization	*In vitro*: MDA-MB453 cells	Incubation with sweet cherry extract enriched in anthocyanins	↓ Sp1 mRNA levels
			↓ Sp4 mRNA levels
			↓ VCAM-1 mRNA levels ↓ Sp1
			↓ Migration ↓ PLCγ-1 ↓ VEGF
			↓ Cell motility Layosa et al. (2021)
Metabolic reprogramming	*In vitro*: PNT1A cells	Incubation with sweet cherry extract	↑ Lactate production ↓ GLUT1 ↓ GLUT3 ↓ PFK-1 ↑ LDH activity ↓ MCT4 Silva et al. (2020)
	*In vivo*: MDA-MB453 cells; xenograft mice mode	Oral administration of sweet cherry extract enriched with anthocyanins	↓ ACAT1 ↓ lipase E, hormone sensitive type Noratto et al. (2020)

4↑ = increase/upregulation; ↓ decrease/downregulation.

### 2.10 *Prunus africana*

*Prunus africana* is a widely distributed tree of African origin which is primarily found in Southern and Central Africa (Komakech et al., 2022). In previous ethnomedical studies, the bark decoction of *P. Africana* was used to treat prostate cancers (Grace et al., 2003; Ochwang’i et al., 2014; Tugume et al., 2016). In *vivo* studies, mice with transgenic adenocarcinoma in their prostate were fed with *P. africana* and demonstrated a significant decrease in the incidence of prostate cancer when compared to control mice fed with casein (Shenouda et al., 2007). Likewise, in human *in vivo* studies, *P. africana* bark extracts induced 50% inhibition of the growth of human prostate cancer (PC-3). It also elicited notable apoptosis *in vitro* in the PC-3 cell line (Shenouda et al., 2007). The anticancer property of the *P. africana* stem bark is attributed to several novel bioactive compounds such as β-amyrin, N-butylbenzene-sulfonamide, β-sitosterol, β-sitosterol-3-O-glucoside, ferulic acid, tartaric acid, oleanolic acid, lauric acid and ursolic acid (Komakech and Kang, 2019). However, *P. africana* is an endangered species and the supply of the *P. africana* stem bark does not meet global demand (Cunningham et al., 2016). A micropropagation protocol has been developed for *P. africana* towards enabling drug development in the future for the treatment of prostate cancer (Komakech et al., 2022). The presence of β-Sitosterol in *P. Africana* has been attributed to its anti-prostate cancer properties wherein it induces apoptosis in human prostate cancer cells during the development of carcinoma in prostate lymph-nodes (von Holtz et al., 1998; Awad et al., 2000). Also, the 2,3-dihydro-3,5-dihydroxy-6-methyl-4H-pyran-one in extracts of *P. africana* is capable of inactivating NF-kB which could account for its proapoptotic and antiproliferative activities on PC-3 cancer cells. Additionally, the occurrence of benzoic acid in these extracts could be responsible for its activity against cancer as earlier studies showed that derivatives of benzoic acid hinder growth of prostate cancer cells thus precluding oncogene expression by the inhibition of histone deacetylases (Anantharaju et al., 2017).

### 2.11 *Securidaca longipedunculata*

*Securidaca longipedunculata* Fresen (violet tree) belongs to the family Polygalaceae, which is called the mother of all medicines in Northern Nigeria. Traditional medical practitioners use extracts of *Securidaca longipedunculata* to manage cancer in Africa (Saidu et al., 2015). The antiproliferative effects of *S. longipedunculata* on Ehrlich ascites carcinoma *in vivo* and *in vitro* are due to the downregulation of angiogenesis and the elicitation DNA fragmentation (Lawal et al., 2012). Root bark extracts of *S. longipedunculata* have been reported to repress proliferation and stimulate apoptosis in U87 brain tumor cells through the cleavage of Poly-ADP-Ribose Polymerase (Ngulde et al., 2019). Ethanolic extracts of the plant hindered the proliferation of the U87 cells. The most polar fraction of these extracts accounted for this activity with an IC_50_ value of 20.535 μg/mL. When administered at 10 mg/kg, the extracts augmented the lifespan of tumor-harboring mice by decreasing tumor cell viability (Ngulde et al., 2019). Several active compounds have been identified in *Securidaca longipendunculata* including xanthones (muchimangins), methyl-salicylate, benzyl benzoates, bisdesmosidic saponins and triterpene saponins, of which xanthones have been implicated in antitumor and cytotoxic activities (Mitaine-Offer et al., 2010; Dibwe et al., 2014; Zuo et al., 2016; Obasi et al., 2018; Klein-Júnior et al., 2020). Saponins in *Securidaca longepedunculata* induced apoptosis and inhibited the migration and invasion of cervical cancer cells (Obasi et al., 2018). Xanthones of in *S. longipedunculata* impeded the proliferation of lung cancer cells and functioned as an elicitor of apoptosis (Zuo et al., 2016).

### 2.12 *Annona senegalensis*

*Annona senegalensis*, which is often called wild soursop, is a 2 to 5-m-tall shrub with alternate, oblong, simple, blue to greenish leaves. It is an important plant in Northern Nigeria and all its parts have been found to be useful in medicine. It contains a plethora of phytochemicals including alkaloids, glycosides, flavonoids, saponins, anthocyanins, tannins, and steroids (Potchoo et al., 2008; Johnson et al., 2017; Musa et al., 2017). Extracts from this plant have been shown to have antitumor activity in hepatocellular carcinoma induced by N-diethylnitrosamine in male Wistar rats. *A. senegelensis* n-hexane extracts have shown anticancer effects by augmenting liver architecture, enhancing antioxidant defense systems, downregulating anti-apoptotic, pro-inflammatory, angiogenic, farnesyl transferase and alpha-fetoprotein mRNA expression and upregulating P21 and P53 tumor suppressor mRNAs (Yakubu et al., 2020).

### 2.13 *Annona muricata*

*Annona muricata* is a tropical fruit tree that belongs to the family Annonaceae. This plant is widely cultivated in African countries such as Angola and some West African countries where its products such as fruits are consumed as food. *Annona muricata* is a plant of great applicability in traditional medicine. Ethanol extracts from the leaves of this plant contain several phytochemicals such as alkaloids, tannins, flavonoids, cardiac glycosides, reducing sugars, triterpenoids and saponins (Gavamukulya et al., 2014). Aqueous extracts of the leaves contain terpenoids, alkaloids, coumarin, flavonoids, fatty acids, steroids, phenols, saponins and tannins while ethyl acetate fractions contain saponins, phenols, flavonoids, polyphenols, and tannins (Agu and Okolie, 2017). *Annona muricata* expresses over 45 acetogenins in its leaves and seeds (Gleye et al., 1998). The ethyl acetate fraction of *A. muricata* leaves induced cytotoxic and antiproliferative activity against breast cancer cells by significantly decreasing mitochondrial membrane integrity, leading to the elicitation of apoptosis in the cells (Hadisaputri et al., 2021). This apoptotic mechanism was identified by alterations in cell morphology and the expression levels of caspase-3, caspase-9 and Bcl-2 mRNAs that were involved in the cytotoxic activity induced by the ethyl acetate fractions on MCF-7 cells (Jabir et al., 2021). prepared silver nanoparticles (AgNPs) by using *A. muricata* as a reducing agent and studying its potential as a novel therapeutic strategy against cancer. Specifically, the ability of these nanoparticles to decrease NLRP3 inflammasome activity by inducing autophagy was examined. The AgNPs showed antiproliferative effects against AMJ-13 and THP-1 cells through the stimulation of apoptosis by damaging the mitochondria and inducing the p53 protein pathway (Jabir et al., 2021). This AgNP-elicited autophagy decreased the levels of IL-1β and NLRP3 inflammasome activation. This study showed that *A. muricata* AgNPs could serve as a robust anticancer agent by inducing apoptosis and autophagy and causing inhibition of tumorigenesis (Eritja et al., 2017; Galluzzi et al., 2017). *Annona muricata* contains two important phytochemicals; acetogenins and flavonoids which have both been implicated in several pharmacological activities (Fang et al., 1993; Yang et al., 2015; Al-Dabbagh et al., 2018). Annonacin, the main acetogenin in *A. muricata*, has anticancer activities against skin, breast and endometrial cancers mediated by cell cycle arrest and the inhibition of other cellular signaling pathways (Yap et al., 2017; Yiallouris et al., 2018; Roduan et al., 2019). There is growing evidence that this antitumor activity is enabled by the induction of apoptosis in several breast and colon cancer cell lines (Rady et al., 2018). Additionally, the G1 phase cell cycle arrest has been reported to be involved in the antitumor activity of *A. muricata* leaf extracts. Both annonacin and ethyl acetate *A. muricata* bark extracts showed selective and robust cytotoxicity against DU-145 prostate carcinoma cells with respective IC_50_ values of 0.1 ± 0.07 μM and 55.501 ± 0.55 μg/mL. Normal RWPE-1 prostate cells were not affected. The chemotherapeutic agent, docetaxel, was, however, devoid of such selectivity. Moreover, when docetaxel was administered in combination with *A. muricata* ethyl acetate bark extracts, its impact against DU-145 cells was enhanced by 50% (Foster et al., 2020). This was attributed to a non-apoptotic mechanism mediating cell death. Annonacin and bark extracts of *A. muricata* acted as selective cytotoxic compounds with antiangiogenic and antimetastatic potential. Leaf methanolic extracts of *A. muricata* (LMAM) showed inhibitory effects on the growth of MCF-7s (Naik and Sellappan, 2020) in a dose-dependent fashion and without causing cytotoxic effects against normal breast cancer cells. This was achieved through an apoptotic pathway (Pieme et al., 2014). Caspase-3 was found to be upregulated, serving as a determinant of apoptosis. LMAM arrested cell cycle at the G1 phase and blocked the G1/S transition attributed to the activation of apoptosis and sub-G0/G1 cell cycle arrest. LMAM displayed major inhibitory activity against MCF-7 cancer cells with an IC_50_ value of 85.55 mg/mL.

### 2.14 *Aerva javanica*

*Aerva javanica* is a shrub that belongs to the Amaranthaceae family of plants. This plant is native to tropical African countries and has been reported to have anticancer properties in Ethiopia (Ayele, 2018). During its use in the treatment of breast cancer, the roots of *A. javanica* are ground into powder and mixed with blood from bats. This mixture is then consumed by the cancer patient early in the morning before breakfast (Teklehaymanot, 2009). The callus and leaf methanol extracts of *A. javanica* were tested against MCF-7s (Teklehaymanot, 2009; Abate et al., 2022) and were found to induce DNA fragmentation and cytotoxicity, which are indicators of apoptosis.

### 2.15 *Abelmoschus esculentus*

*Abelmoschus esculentus*, also known as Okra, is a native African plant of the family Malvaceae. It has been historically been used to treat different ailments such as constipation, hypoglycaemia, inflammation and microbial infections (de Sousa Ferreira Soares et al., 2012). The seeds of *A. esculentus* contain flavonoids such as isoquercitrin which have shown cytotoxicity towards carcinoma cell lines; MCF-7 (breast cancer), HepG2 (liver cancer) and HeLa (cervical cancer) (Agregán et al., 2022). Okra flower extracts, which are rich in flavonoids, are capable of inhibiting cell proliferation in colorectal tumors and this inhibitory activity has been found to be due to the dysfunction of the mitochondria which is caused by the activation of p53 and the elicitation of apoptosis and senescence. In mice models, flavonoids in *A. esculentus* have shown cancer preventative activities which precluded tumor appearance. Okra flower phytochemicals positively impacted liver cancer prognosis while Okra ethyl acetate extracts showed inhibitory activity against HepG2 cells at concentration ranges between 62.5 and 1,000 μg/mL (Solomon et al., 2016). Therefore, Okra flowers were shown to be a valuable source of anticancer molecules which could augment the health of cancer patients. A novel lectin has been found in Okra seeds and it promotes antitumor effects in MCF-7s wherein it inhibits cell growth. It also enhances p21, caspase-3 and caspase-9 expression in the carcinogenic cells (Monte et al., 2014). Cerium oxide (CeO_2_) nanoparticles were generated using *A. esculentus* as a stabilizing and reducing agent. Exposure of HeLa cells to these CeO_2_ nanoparticles at 10–125 μg/mL resulted in the loss of cell viability in the cervical cancer cells in a dose-dependent fashion (Ahmed et al., 2021). *Abelmoschus esculentus* has been shown to exhibit anticancer effects due to its high antioxidant activity against free radicals (Arapitsas, 2008; Ahmed and Kumar, 2016). It is a source of polysaccharides, flavonoids, terpenoids, tannins, alkaloids, enzymes, proteins, and vitamins (Musthafa et al., 2021) demonstrated the apoptotic potential of *A. esculentus* lectins against human glioblastoma cells by the modulation of the caspase-3 and caspase-7 gene expression and the downregulation of CLOCK and Bma1 circadian genes, implicating a correlation between these circadian genes and apoptotic cell death. Increased cytotoxicity, morphological changes, increased intracellular ROS generation and anti-migratory activity were also observed. Due to these effects, *A. esculentus* extracts can be considered as adjunct therapy in alleviating human glioblastoma. Furthermore, pulp extracts of *A. esculentus* were used to synthesize gold nanoparticles which showed enhanced anticancer effects (Devanesan and AlSalhi, 2021). Silver nanoparticles made using *A. esculentus* flower extracts showed antiproliferative, apoptotic and cytotoxic effects against A-549 and TERT-4 cancer cell lines. Quercetin diglucoside and isoquercitrin identified in *A. esculentus* were shown to be potently anti-ROS and thus could be used in cancer treatment (Taiwo et al., 2021).

### 2.16 *Flueggea virosa*

*Flueggea virosa* is widely distributed is the Southern Africa. *Flueggea virosa* leaves and twigs were used to isolate flueggines A 1) and B 2) indolizidine alkaloids, the latter of which showed notable inhibitory activities against the growth of breast cancer cells MDA-MB-231s (estrogen-independent) and MCF-7s (estrogen-dependent) with IC_50_ values of 147 ± 3 and 135 ± 5 nM respectively. This suggested that cell proliferation was inhibited irrespective of estrogen receptor status (Zhao et al., 2011).

### 2.17 *Lagenaria siceraria*

*Lagenaria siceraria* is a native African climbing plant that belongs to the Cucurbitaceae family (Abebe, 2016). (Mondal and Swamy, 2020) identified BGL24, a novel PP2-type lectin in the phloem exudate of this plant, which is also called Bottle gourd. This lectin displayed high specificity for chito-oligosaccharides. BGL24 showed moderate cytotoxicity towards MDA-Mb-231 breast cancer cells but did not impact normal splenocytes. The latex sap of *L. siceraria* (LSL) markedly elicited lymphocyte proliferation and demonstrated potent cytotoxicity against cancer both *in vivo* and *in vitro* (Vigneshwaran et al., 2016). LSL caused tumor regression and drastically impacted tumoral neovasculature. Additionally, LSL stimulated the apoptotic signaling cascade in tumor cells through the activation of caspase-3 and the induction of apoptotic cellular events. Therefore, LSL possesses immunopotentiating properties which negatively impact tumor progression by attacking angiogenesis and inducing programmed cell death, which are major hallmarks of cancer. Bottle gourd juice (BGJ) was examined for its chemopreventive properties against croton oil and 7,12-dimethylbenz(a)anthracene (DMBA) and was found to elicit skin papillomagenesis in murine models (Kumar et al., 2013). BGJ caused drastic reductions in the incidence, latency, number, multiplicity, size, and volume of the papillomas. This chemopreventive effect was mediated by reducing loss of stratification, decreasing the number of epithelial layers, diminishing dermal infiltration and protection from several cytoplasmic changes. Therefore, BGJ consumption could help in the inhibition of skin cancer.

### 2.18 *Xylopia aethiopica*

Cytotoxic metabolites have been identified in methanol extracts of *X. aethiopica* and were found to inhibit the growth of multidrug resistant and drug-sensitive cancer cell lines (Kuete et al., 2015). Among these metabolites, flavone elicited apoptosis in CCRF-CEM leukemia cells through the disruption of the mitochondrial membrane potential and isoquinoline triggered apoptosis *via* the production of ROS, implicating these compounds as antiproliferative agents against drug-resistant cancers. Essential oils obtained from *Xylopia aethiopica* sourced from Cameroon and Chad were found to be abundant in monoterpene hydrocarbons such as β-phellandrene, β-pinene, γterpinene and sabinene. Oxygenated monoterpenes were highly prevalent, amongst which terpinen-4-ol was most significant (Bakarnga-Via et al., 2014). Hydroethanolic extracts of *X. aethiopica* showed antiproliferative activity against HCT116 colon cancer cells as well as KG1a and U937 leukemia cell lines. *Xylopia ethiopica* extracts also demonstrated antiproliferative effects on human cervical cancer cells. Furthermore, α-cadinol and terpinen-4-ol in essential oils isolated from *X. ethiopica* were found to be active against laryngeal, lung, ovarian, breast, gastric and colon cancer cell lines (Bakarnga-Via et al., 2014). Additionally, βpinene, a monoterpene found in this oil, showed notable cytotoxic activity against epidermal skin and breast cancer cell lines. Fruit extracts of *X. aethiopica* caused the activation of caspase-3 and led to the cleavage of cytoskeletal proteins and the elicitation of DNA fragmentation factors, condensation of chromatin, formation of apoptotic bodies and eventually apoptosis as observed through morphological analysis (Fulda and Debatin, 2006; Ribeiro et al., 2021). (Adaramoye et al., 2011) showed that fruit extracts of *X. aethiopica* caused antiproliferative activity against human cervical carcinoma cells causing cell cycle arrest and elevated levels of p53 and p21 gene transcripts. Caspase-3 activation and apoptotic cell death have been demonstrated in MBA-MD-231 breast cancer cells upon exposure to hydroethanol extracts sourced from the Cameroonian varieties of *X. aethiopica* fruits (Choumessi et al., 2012).

### 2.19 *Nymphaea lotus*

*Nymphaea lotus* belongs to the family Nymphaeaceae and is a perennial aquatic flowering plant that is native to Egypt and grown in several regions in Madagascar, West Africa, and Central Africa (Slocum, 2005). Hydroethanolic leaf extracts of *N. lotus* contain saponins, tannins, flavonoids, phenolics and triterpenoids but are lacking in alkaloids. These extracts have been shown to have anti-inflammatory and cytotoxic activities along with high antioxidant potential against Jurkat and MCF-7s, properties that are attributed to the occurrence of abundant flavonoids and phenolics as well as micro/macro-elements such as sulfur, phosphorus, manganese, magnesium, zinc and copper (Saleem et al., 2001; Elegami et al., 2003; Laszczyk, 2009; Afolayan et al., 2013; Oyeyemi et al., 2015; N’guessan et al., 2021). These properties may account for the use of the leaves of *Nymphaea lotus* in traditional anticancer treatments.

### 2.20 *Zanthoxylum chalybeum*

*Zanthoxylum chalybeum* is a deciduous shrub with a rounded but open crown. It can grow between 1.5 and 40 cm in diameter, with large woody spines that can grow up to 2 cm long. It is a widely used traditional medicine in East Africa and can be harvested from the wild for local use as tea, medicine, toothbrush, and timber. *Zanthoxylum chalybeum* is reported to exhibit anti-cervical cancer properties, where part of the plant used is pound before adding water to drink (Tugume et al., 2016; Omara et al., 2020). Significant antiproliferative effects of crude extracts of alkaloids from *Zanthoxylum* species were observed against human cervical cancer cells (HeLa), human gastric cancer cells (SGC-7901), human hepatocyte carcinoma cells (Hep G2) and human colorectal adenocarcinoma cells (HT29), ranging from 60.71% to 93.63% at 200 μg/mL concentration (Tian et al., 2017). *Zanthoxylum* contains quaternary alkaloids which have been shown to be potential anti-cancer candidates that can penetrate through the cell membranes of carcinomas (Yung et al., 2016) and can attract the negative charges on DNA (Bai et al., 2006). Based on their structural skeleton, these quaternary alkaloids have been shown to belong to the berberine type, tetrahydroproberberine type, benzophenanthridine type, benzyltetrahydroisoquinoline type and aporphine type (Tian et al., 2017). In *Z. chalybeum*, the amount of quaternary alkaloids account for 83.4%. Bioactivity tests on *Zanthoxylum* showed both high inhibitory rates against cancer cells and high quaternary alkaloid content, therefore justifying its use in traditional anticancer medications. The active ingredients responsible for the anticancer activity of this plant include skimmianine, furoquinoline, benzophenanthridine, alkaloids, chelerythrine and nitidine, aporphine alkaloids, tembetarine, N-methylisocorydine, N- methylisocorydine (menisperine) and bernerine, phenylethylamine, candicine, alkamide, fagaramide, dihydrochelerythrine, lupeol and sesamin (Omara et al., 2020).

### 2.21 *Ceratonia siliqua*

*Ceratonia siliqua* is a Mediterranean evergreen plant that is abundantly distributed in North African countries such as Algeria, Morocco, Tunisia, and Egypt (Kaderi et al., 2015). Common ethnomedicinal uses of *Ceratonia siliqua* include treatment of gastrointestinal diseases, diarrhea, constipation and colon cancer (Benarba and Pandiella, 2018). These medicinal properties may chiefly be due to the presence of fibers and phenolic compounds (Rtibi et al., 2017). (Ghanemi et al., 2017) demonstrated that *C. siliqua* leaf phenolic extracts inhibited the growth of HTC-116 and CT-26 cell lines, in a dose dependent manner, confirming earlier similar findings by (Custódio et al., 2013).

### 2.22 *Moringa oleifera*

*Moringa oleifera*, a member of the Moringaceae family, is a drumstick tree which was first used medically by the ancient Egyptians before its cultivation around the globe which spread its medicinal benefits (Abd-Rabou et al., 2017). *Moringa oleifera* was found to be effective in treating colon cancer (Al-Asmari et al., 2015). The plant owes its anticancer properties to the presence of quercetin, kaempferol, β–D-glucopyranoside, tetracanoate, β–sitosterol glucoside, isothiocyanate (Kaur, 2015), hexadecenoic acid and eugenol (Al-Asmari et al., 2015). (Suphachai, 2014) reported the growth inhibition of hepatocarcinoma (HepG2), colorectal adenocarcinoma (Caco-2), and breast adenocarcinoma (MCF-7) cell lines by dichloromethane leaf extracts of *Moringa oleifera* with IC_50_ values between 112 and 113 μg/mL. Recently, *in vitro* in *vivo* anticancer activities of *M. oleifera* have been reported. In their study (Barhoi et al., 2021), identified quinic acid, octadecanoic acid and hexadecanoicacid (palmitic acid) as the active compounds during the activity of aqueous extracts of *M. oleifera* on Ehrlich ascites carcinoma (EAC) and Hep2 (Human laryngeal carcinoma) cells. They reported that, *in vivo*, aqueous extracts of *M. oleifera* led to a reduction in tumor weight and tumor volume in tumor bearing mice, consequently elongating the life span of the mice. Additionally, *in vitro*, the extracts of *M. oleifera* were toxic to both Hep2 and EAC cancer cell lines. Apoptosis was also induced through the alteration of the mitochondrial membrane potential in the EAC cells (Barhoi et al., 2021).

It has also been recently shown that methanolic extracts of *M. oleifera* leaves led to a reduction in cell growth in cervical cancer cells (Pandey and Khan, 2021). The cervical cancer cells were reported to have undergone apoptosis. The anticancer potential of the *M. oleifera* leaf extracts was due to the inhibitory activity of the extracts on Jab-1, which is an important biomarker associated with the development of different cancers (Pandey and Khan, 2021). The inhibition of *M. oleifera* on Jab-1 led to its downregulation. There was also an increase in the expression of the tumor suppressor p27, which led to cell growth arrest at the G0/G1 phase.

Methanol extracts of *M. oleifera* leaves have also been reported to have apoptotic effects on PC-3 prostate cancer cells (Khan et al., 2020). The anticancer activity of *M. oleifera* leaf methanolic extracts was due to the induction of ROS-mediated apoptosis and the activation of caspase-3 activity in the prostate cancer cells. Cell cycle arrest at the G0/G1 phase and changes in the expression of genes of the Hedgehog signalling pathway were also observed (Khan et al., 2020).

*Moringa oleifera* leaf extracts have been shown to have anticancer activity on the human squamous cell carcinoma 15 cell line (SCC15). In their study (Luetragoon et al., 2020), reported that the proliferation of SCC15 cells treated with *M. oleifera* leaf extracts was inhibited. The extracts were shown to induce cell cycle arrest at the G2/M phase and apoptosis in the cells. Cell migration and colony formation were also inhibited in the cells. Furthermore, there was a downregulation of the anti-apoptotic marker Bcl-2 and an upregulation of both Bax and caspase-3 (Luetragoon et al., 2020).

Through the activity of its bioactive compound 4-[(α-L-Rhamnosyloxy) benzyl] isothiocyanate (MIC-1), which is found in the seeds, *M. oleifera* has been reported to inhibit the migration and proliferation of renal cancer cells (Xie et al., 2022). This regulatory activity is said to be brought about by the regulation of the PTP1B-dependent Src/Ras/Raf/ERK signalling pathway in 786-O hypertriploid renal cell carcinoma (RCC) cancer cells. MCI-1 from *M. oleifera* seeds also induced cell cycle arrest and caused the downregulation of the expression of cell cycle-related proteins in the 786-O cells (Xie et al., 2022).

### 2.23 *Peganum harmala*

*Peganum harmala* is a perennial herbaceous plant with a woody underground root stalk. It is a member of the Nitriaceae family and mostly grows in temperate deserts and Mediterranean regions (Alves-Silva et al., 2017). reported the ethnomedicinal uses of *P. harmala* as a remedy for the treatment of breast, liver, and bone cancer. It is also considered as a treatment for other different types of cancer (Kabbaj et al., 2012). *Peganum harmala* seeds from Morocco are ground with honey during cancer treatment. Despite the ethnomedicinal uses of *P. harmala*, the plant is also toxic and may cause hallucinogenic effects because of the presence of β-carbolines such as harmaline, harmine, harmalol, harmol, tetrahydroharmine, and the quinazoline derivatives vasicinone and deoxyvasicinone (Passos and Mironidou-Tzouveleki, 2016). Table 3 gives a summary of some of the *in vivo* and *in vitro* studies that has been carried out on plants that have been discussed in the manuscript as well as the identified bioactive compounds in these plants and the effects of these compounds on cancer progression.

**TABLE 3:** A summary of pharmacological information of all the medicinal plants that have been discussed in the manuscript.

Plant species	Bioactive compounds	Cell line/Experiment	Effects	References
*Dicoma anomala*	Acetylenic compounds, flavonoids, phenolic acids, phytosterols, sesquiterpenes and triterpenes	MCF-7 breast cancer cells	Reduction of proliferation, oxidative damage of the cells	Shafiq et al. (2020)
*Fagaropsis angolensis*	Alkaloids, glycosides, phenols, tannins, steroids, and flavonoids	Hep2 throat cancer cells, CT 26-CL 25 colon cancer cells	Antiproliferation of cell lines, induction of programmed cell death by apoptotic pathways	Yiaile (2017)
*Tribulus terrestris*	Saponin compounds such as nuatigenin saponin	TW2.6 and SAS oral cancer cells	Inhibition of autophagy, inhibition of cell growth and proliferation, invasion of neoplastic cancer cells	Shu et al. (2021)
*Portulaca oleracea*	POL-P3b, Glycosides such as oleraciamide E and oleraciamide F	Mice models	High level induction of TNF-α, IFN-γ and IL-12, tumor growth inhibition, induction of apoptosis	Jia et al. (2021)
*Withania somnifera*	Withaferin A and Withanolide D	B16F-10 melanoma cells in C57BL/6 mice	Significant inhibition of tumor activity	Leyon and Kuttan (2004)
*Azanza garckeana*	Mansone G, β-Cyclodextrin	A549 lung cancer cells	High toxicity towards the cells	Bioltif et al. (2020)
*Cajanus cajan*	Stilbenes longistylins A and C, β-sitosterol, pinostrobin	COR-L23 lung cancer cells	Cytotoxicity towards the cells, cell growth arrest	Ashidi et al. (2010)
*Combretum caffrum*	Combrestatins such as combrestatin A-4 (CA-4)	Leukemia cell line, P-388	Disruption cell signaling pathways, selective disruption of blood flow through tumors	McGown and Fox (1990)
*Prunus avium*	Phenolics, anthocyanins	Wistar rats	Inhibition of lipid peroxidation, decrease in catalase	Dziadek et al. (2019)
*Prunus africana*	Alkaloids, glycosides, phenols, tannins, steroids, and flavonoids	Hep2 throat cancer cells, CT 26-CL-25 colon cancer cells	Antiproliferative effect against the cells	Yiaile (2017)
*Securidaca longipedunculata*	Benzyl benzoates, bisdesmosidic saponins and triterpene saponins	U87 brain tumor cells	Inhibition of proliferation, induction of apoptosis	Ngulde et al. (2019)
*Annona senegalensis*	Alkaloids, glycosides, flavonoids, saponins, anthocyanins, tannins, and steroids	Male Wistar rats	Augmentation of liver architecture, upregulation of p21 and p53	Yakubu et al. (2020)
*Annona muricata*	Terpenoids, alkaloids, coumarin, flavonoids, fatty acids, steroids	MCF-7 breast cancer cells	Morphology alterations, induction of apoptosis	Jabir et al. (2021)
*Aerva javanica*	Phenolics, flavonoids, lignins, terpenes, glycosides, and alkaloids	MCF-7 breast cancer cells	Antiproliferation, apoptosis induction through DNA fragmentation and cytotoxicity towards the cells	Abate et al. (2022)
*Abelmoschus esculentus*	Flavonoids such as isoquercitrin	HepG2 liver cancer cells	Cytotoxicity towards the cells	Agregán et al. (2022)
*Flueggea virosa*	Flueggines A 1) and B 2) indolizidine alkaloids	MDA-MB-231 breast cancer cells	Growth inhibition of the cells, inhibition of proliferation	Zhao et al. (2011)
*Lagenaria siceraria*	PP2-type lectin BGL24	MDA-MB-231 breast cancer cells	Moderate toxicity towards the cells	Mondal and Swamy (2020)
*Xylopia aethiopica*	Flavonoids, alkaloids	CCRF-CEM leukemia cells	Induction of apoptosis, disruption of the mitochondrial membrane potential, production of ROS	Kuete et al. (2015)
*Nymphaea lotus*	Saponins, tannins, flavonoids, phenolics and triterpenoids	MCF-7 breast cancer cells	Anti-inflammation, cytotoxicity, high oxidant activity	N’guessan et al. (2021)
*Zanthoxylum chalybeum*	Alkaloids, furoquinoline, benzophenanthridine, alkaloids, chelerythrine and nitidine, aporphine alkaloids, tembetarine, N-methylisocorydine	HeLa cervical cancer cells	Antiproliferative effects on the cells	Omara et al. (2020)
*Ceratonia siliqua*	Phenolic compounds	HTC-116 and CT-26 colon cancer cell lines	Growth inhibition	Ghanemi et al. (2017)
*Moringa oleifera*	Quercetin, kaempferol, β–D-glucopyranoside, tetracanoate, β–sitosterol glucoside, isothiocyanate	MDA-MB-231 breast cancer cells and HCT-8 ileocecal cancer cells	Reduction in cell survival, reduction in colony formation, increase in apoptosis	Al-Asmari et al. (2015)
*Peganum harmala*	Phenols, tannins, flavonoids and anthocyanins	RT112 human bladder carcinoma cells	Antioxidant activity	Al-Asmari et al. (2015)

## 3 Semi-synthetic studies for new drugs derived from natural compounds for optimizing anticancer activity

Over 25% of drugs used for combating human diseases are directly sourced from plants and another 25% are chemically modified natural products (Amin et al., 2009). Polyphenols constitute a plethora of naturally occurring compounds found in vegetables and fruits. Their health-enhancing characteristics and their application in the prophylaxis and therapy of many human cancers are well known. Several anti-cancer drugs are altered forms of these natural compounds. Etoposide (VP-16, epipodophyllotoxin) is an anticancer drug derived semi-synthetically from a non-alkaloid lignan, podophyllotoxin sourced from the rhizomes and dried roots of *Podophyllum emodi* or *Podophyllum peltatum* (Kluska and Woźniak, 2021). Etoposide has been widely employed in cancer chemotherapy to combat various types of cancer such as adrenocortical carcinoma (Paragliola et al., 2020), brain tumors (Ruggiero et al., 2020), breast cancer (Atienza et al., 1995), leukemia (Economides et al., 2019), testicular carcinoma (Alsdorf et al., 2019) and small cell lung carcinoma (Noronha et al., 2020).

Polyphenols enhance the therapeutic action of etoposide by augmenting its cytotoxicity in various cancer cell lines like the retinoblastoma (Sreenivasan and Krishnakumar, 2015), glioblastoma (Ramachandran et al., 2012), breast cancer (Ermakova et al., 2006), cervical cancer (Ruíz et al., 2018), liver cancer (Jia et al., 2016), gastric cancer (Yu et al., 2011; Jia et al., 2016), osteosarcoma (Ferreira de Oliveira et al., 2018), lymphoma (Noda et al., 2007; Li et al., 2008), colon cancer (HWANG et al., 2007; Amiri et al., 2013), head and neck cancer (Heiduschka et al., 2014) and leukemia (Mahbub et al., 2015; Papież et al., 2016). This effect of polyphenols is caused by an increase in DNA damage and apoptosis, production of ROS and arrest of the cell cycle. Investigations on the human breast cancer cell line, MDAMB-231 revealed that flavonoids such as cyanidin, fisetin, (−)-catechin, kaempferol, naringenin, genistein and quercetin, blocked the DNA damage checkpoints and the respective repair pathways. These polyphenols inhibited Chk1 Ser345 phosphorylation induced by etoposide, and this led to the abrogation of the ATR-Chk1 pathway (Kuo et al., 2016). Hence, polyphenols can enhance the therapeutic action of etoposide chemotherapy by rendering the cancer cells more drug sensitive (Fiorito et al., 2014). synthesized a select group of natural and semi-synthetic 1,4-Naphthoquinones whose activity in inhibiting cell growth was studied *in vitro* on six human cancer cell lines. Among these compounds, only lapachol and its acetate as well as 3-geranyllawsone showed the highest activity with 15–22 μM IC_50_ values. Several novel anticancer drugs currently under commercial production are derived from natural plant sources and these include etoposide, irinotecan, topotecan, taxotere, taxol, teniposide, vinorelbine, vincristine and vinblastine (Wang, 1998). Thus, natural products serve as the most important source of novel anticancer agents.

Therefore, 1,4-Naphthoquinones constitute a major category of natural products found in plants. Plumbagin (obtained from *Plumbago, Nepenthes Drosera spp*.), juglone (sourced from the black walnut, *Juglans nigra L*. (Juglandaceae), and the K vitamins are significant examples of these 1,4-Naphthoquinones. The action mechanisms underlying these observed effects are primarily due to their ability to react with topoisomerases and to produce semiquinone radicals as well as ROS inside the cell (da Silva et al., 2002). (Kode et al., 2020) synthesized Compound 9a containing 1-methyl, 2- and 3-methoxy substituents within the aromatic ring of phenstatin based indole linked chalcone compounds. This was found to be effective against the SCC-29B human oral cancer cell line, spheroids as well as AW13516, an oral cancer mouse xenograft model. Compound 9a anticancer activity was found to be effected by the disruption of glucose metabolism and cellular integrity wherein the latter was caused by repression of tubulin polymerization. Compound 9a was found to directly interact with tubulin at the colchicine binding site in addition to interacting with the active sites of key enzymes involved in the pathway of glucose metabolism (Kode et al., 2020). Thus, compound 9a has found great applicability as a favorable tubulin polymerization inhibitor candidate in anti-cancer therapeutics. Compound 9a greatly reduced the tumor volume while not causing any toxicity in murine models. Additionally, it decreased angiogenesis in xenografts of mice, diminished collagen levels and significantly reduced cellular processes including cellular integrity and organization of the cytoskeleton, while disrupting the uptake of glucose in tumor xenografts. The tubulin polymerization was inhibited which led to profilament bending, destabilization and formation of tubulin ring intermediates.

Lupeol (LU) and Oleanolic acid (OA) are members of the class of natural triterpenes and possess wide-ranging biological activities and cytotoxicity against many cancer cell lines. From these two compounds, 6 novel semi-synthetic triterpenes were synthesized and studied for their pharmacological action (Fontana et al., 2022). Compared to its precursor, Lupeol, the lupane-like compounds showed enhanced activity whereas the oleane-like compounds exhibited more complex properties. Both LU and OA derivatives displayed a pattern of interaction with the NF-κB p65 subunit that justified the similarity in their capability to hinder the p65 binding to DNA. Also, some of the tested derivatives could augment IκB-α levels precluding the NF-κB translocation to the nucleus. This study revealed the pharmacological activity of triterpenes towards leukemia cells, making them valuable anticancer drug candidates.

The different OA and LU derivatives were investigated for their cytotoxic effects against acute myeloid leukemia cancer cell line HL60 as well as its multidrug-resistant (MDR) variant HL60R wherein the latter displayed multidrug resistance, P-gp overexpression, constitutive expression of the NF-κB transcription factor, inhibition of proteins associated with apoptosis and poor prognosis (Fontana et al., 2022). These derivatives showed more activity than the original compounds and displayed equivalent cytotoxic effects against both HL60 and HL60R cell lines. Also, these compounds remarkably disrupted the NF-κB pathway both downstream and upstream to NF-κB activation within the MDR cell line.

All these compounds were able to hinder transcription factor transactivation in the HL60R cell line. Both LU and OA derivatives displayed similar patterns of interaction with the NF-κB p65 unit, that justified the similarity in their potential to inhibit the interaction of p65 to DNA as well as some of its molecular targets. The capability of many of the derivatives to augment IκB-α levels precludes the transcription factor from movement to the nucleus, thus accounting for the indirect effect of NF-κB inhibition. This investigation showed that pre-treatment with OA and LU derivatives could potentiate increased sensitivity of the cancer cells to standard drugs used in chemotherapy, thereby representing novel therapeutic approaches to combat acute myeloid leukemia.

Terpenoids are the largest class of natural compounds and are known for their anticancer activity. (Hussain et al., 2018), explored the anticancer effects of a novel semi-synthetic terpenoid, 16-oxo-cleroda-3,13 (14)E-diene-15-oic acid 1), derived from cleroda diterpene isolated from *Polyalthia longifolia* var. *pendula* against neuroblastoma (Faizi et al., 2008). The γ-amino γ-lactone (PGEA-AN, 2) of one was chosen for further study as it displayed the highest cytotoxic activity upon initial screening.

PGEA-AN was shown to modulate the p53 system resulting in death of the neuroblastoma cells while not affecting the renal system making it a promising candidate for anticancer activity against neuroblastoma. Further, PGEA-AN augments mitochondrial membrane permeability (MMP). Induction of P53 in neuroblastomas is common observation associated with chemotherapeutic agents (Nikolaev et al., 2003; Liontas and Yeger, 2004). Notwithstanding the mode of cell death, p53 has been implicated in the mitochondrial pathway leading to cell death resulting from apoptosis or necrosis (Cui et al., 2002; Vaseva et al., 2012). The p53 gene directly upregulates BAX, a proapoptotic gene (Toshiyuki and Reed, 1995). BAX overexpression leads to augmentation of apoptosis mediated by multiple factors (Kobayashi et al., 2000). Activation of BAX results in its translocation into mitochondria which in turn increases MMP (Youle and Strasser, 2008), causing reduction or loss of mitochondrial transmembrane potential, pores in the mitochondrial membrane and promotes necrotic or apoptotic cell death (Tsujimoto and Shimizu, 2007).

*Peganum harmala* L is a medicinal plant of great importance due to its plenitude of alkaloid content rich in ß-carbolines (Daoud et al., 2014). report the anticancer activity of a semi-synthetic derivative, B-nine to three formed from two harmane molecules that are bound by a butyl group. This compound showed strong potency and anti-proliferative effects against a human colorectal carcinoma cell line, a human breast cancer cell line and a human lung cancer cell line. A dose-dependent elicitation of apoptosis or necroptosis was observed against all these cell lines in addition to repression of cancer cell migration. Also, B-nine to three exhibited anti-angiogenic effects *in vitro* as observed by the drug-mediated inhibition of tube formation within the human umbilical vascular endothelial cell line (HUVEC).

As per epidemiological findings, chronic inflammation has been implicated in 25% of cancer cases. Hence, blockage of carcinogenesis induced by inflammation could be a viable therapeutic approach for the chemoprevention of cancer. Moreover, anti-inflammatory drugs have been shown to decrease the incidence of cancer. Saponins are natural compounds having tumor inhibitory properties. The triterpenoid derivative of oleanolic acid, 2-cyano-3, 12-dioxooleana-1, 9 (11)-dien-28-oate (CDDO) inhibited NFκB signaling and displayed antitumoral and anti-inflammatory activities, authenticating its therapeutic potential (Place et al., 2003; Shishodia et al., 2006). This effect was also substantiated in murine models of prostate cancer (Gao et al., 2011).

Semi-synthetic analogs of cycloartane-type sapogenols (9,19-cyclolanostanes) were prepared (Debeleç-Bütüner et al., 2018) and five of these analogs were investigated for their anticancer activity. Of these, astragenol derivatives 1 and 2, and the cycloastragenol derivatives 3, 4, and 5 showed strong inhibition of NFκB signaling resulting in the blockage of NFκB transcriptional activation and suppression of cell proliferation. Therefore, these semi-synthetic compounds displayed strong potential for chemoprevention of prostate cancer driven by the inflammatory NFκB signaling pathway.

## 4 Conclusion

Several synthetic drugs have been used to treat cancer but cause long term effects. This review provides an update on advances in the use of secondary metabolites from different African medicinal plants with anticancer potential on different biological models including human cell lines, animals, and human models. Most of the studies that we assessed employed *in vitro* assays with some *in vivo* assays. We report some plants that have contributed to cancer management in Africa, where the anticancer extracts are usually prepared from bark, fruits, leaves, roots, and stems. *In vitro* and *in vivo* studies mentioned in this review have shown that bioactive compounds and metabolites from African medicinal plants use a variety of mechanisms during cancer management. Carrying out *in vitro* studies on anticancer plants is a very important initial step in pharmacological testing. Testing plant phytochemicals on cancer cell lines allow scientists to gauge the anticancer potential of these phytochemicals in a safe and controlled environment of the lab. *In vitro* studies also allow testing of different combinations of phytochemicals on cancer cells to determine any synergistic advantages. Once the effects of the phytochemicals on cancer cells are determined, the phytochemicals are then tested *in vivo* using model organisms such as mice and rats. After the efficacy of the phytochemicals is confirmed *in vivo*, pre-clinical studies are then undertaken to test the phytochemicals on a small population of clinical candidates. If successful, and after approval by appropriate regulatory bodies, the phytochemicals, in drug form, can then be clinically applied in cancer management. Therefore, *in vitro,* and *in vivo* studies of potential anticancer phytochemicals from plants play a crucial role in the ultimate clinical application and commercialization of phytochemicals of these plants, providing alternative cancer management strategies to mankind. However, there is insufficient research on other African medicinal plants for potential use in cancer management. Therefore, there is a need to isolate and evaluate the anticancer potential of the bioactive compounds in the understudied medicinal plants of Africa and elucidate their mechanisms of anticancer activity. Furthermore, more research on animal models is required as it can lead to more clinical studies. Despite the anticancer potential of the reviewed African medicinal plants, there is insufficient data on *in vivo* studies and targets of cancer. *In vivo* studies are crucial for pre-clinical studies and ultimate drug development. Furthermore, toxicological evidence is insufficient in some of the mentioned studies. In some populations, some medicinal plants may be allergenic or harmful, while in other cases, specific plant parts may either be edible or poisonous. Therefore, toxicology investigations are necessary for the determination of adverse effects of some of the plant extracts. They are also important in the establishment of limits of exposure levels.

**FIGURE 2:**
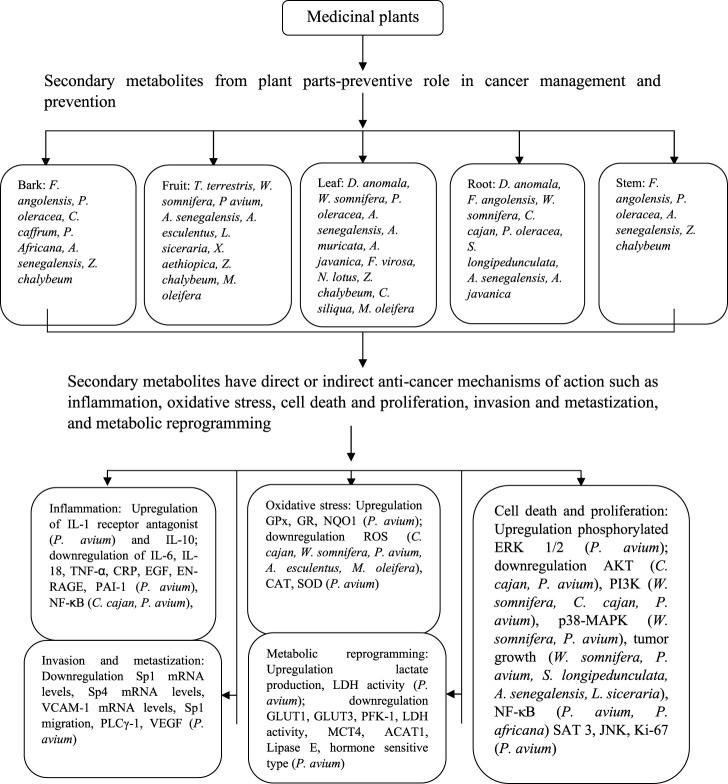
Diagrammatic depiction of medicinal plant parts involved in therapeutic effects against various types of cancers and their target pathways.

Many genes, proteins and signaling pathways play important roles in cancer development, progression, and alleviation. Based on the findings of this manuscript, the following genes and pathways should be investigated for preclinical studies as they are significant to cancer biology; the tumour suppressors p21 and p53 as well as the following pathways: NF-κB, AKT, PI3K, p38-MAPK pathways. In summation, the take home message of this review is that African plants have been shown to have potential anticancer activity. It is important that *in vitro* studies mentioned in this review are advanced to *in vivo* investigations and the *in vivo* studies are elevated to pre-clinical studies. This review has collated information that may be critical in guiding researchers interested in exploring native African plants for use in cancer management. Figure 2 summarize the different pathways on which different plants their compounds and extracts.

## Glossary

ACAT1: Acetyl-CoA acetyltransferase
Akt: Ak strain transforming serine/threonine protein kinase
ASC adaptor protein: Apoptosis-associated Speck-like protein containing a CARD BGL24 bottle gourd Lagenaria siceraria
Bcl-2: B-Cell Leukemia/lymphoma-2
Bim: BH3-containing protein
Con A: Concanavalin A
COX-2: CycloOXygenase-2
CRP: C-Reactive Protein
DC: Dendritic Cell
EGF: Epidermal Growth Factor
ENRAGE: Extracellular Newly identified ligand for the Receptor for Advanced Glycation End products
ER: Estrogen Receptor
ERK: Extracellular signal-Regulated Kinase
FOXO3a: Forkhead box O3a
GLUT: glucose transporter
GPx: glutathione peroxidase
GSH: reduced glutathione
GSSG: Glutathione Disulfide GR Glutathione Reductase
H2O2: Hydrogen peroxide
HSF1: Heat Shock Factor
IC50: Inhibitory Concenration50
IFN-γ: Interferon-γ
ING1: Inhibitor of Growth family, member1
IL-12: Interleukin-12
JNK: c-Jun N-terminal kinase
LDH: Lactate dehydrogenase
LHX3: LIM Homeobox 3
LPS: Lipopolysaccharide
MAPK: Mitogen-Activated Protein Kinase
MCT4: MonoCarboxylate Transporter 4
MyD88: Myeloid Differentiation primary response 88
NCAM: Neural Cell Adhesion Molecule
NF-κB: Nuclear Factor-κB
NLRP3: NLR family Pyrin domain containing three
NO synthase: Nitric Oxide synthase
NQO1: NADPH quinone oxidoreductase
PI3K: Phosphoinositide three Kinase
PAI-1: Plasminogen Activator Inhibitor-1
PFK-1: Phosphofructokinase-1
PLCγ-1: Phosphoinositide-specific Phospholipase γ1
PP2: Phloem Protein 2
RET: REarranged during Transfection tyrosine kinase
ROS: Reactive Oxygen Species
SCID: Severe Combined Immunodeficiency
SOD: SuperOxide Dismutase
Sp1/Sp4: Specificity protein transcription factors
STAT3: Signal Transducer and Activator of Transcription three
TFAP2A: Transcription Factor AP-2 alpha
Th1: T helper cell
TLR4: Toll-Like Receptor4
TNF-α: Tumor Necrosis Factor-α
TPX2: Microtubule-associated Protein
TUNEL: Terminal deoxynucleotidyl transferase TdT-catalyzed dUTP-Nick End Labeling
VCAM-1: Vascular Cell Adhesion Molecule-1
VEGF: Vascular Endothelial Growth Factor
